# Glutamate as a Therapeutic Substrate in Migraine

**DOI:** 10.3390/ijms26073023

**Published:** 2025-03-26

**Authors:** Nazia Karsan, Luiza Bastos Alves, Peter J. Goadsby

**Affiliations:** 1 Headache Group, Wolfson Sensory, Pain and Regeneration Centre (SPaRC), Institute of Psychiatry, Psychology and Neuroscience, King’s College London, London SE5 9PJ, UK; nazia.karsan@kcl.ac.uk (N.K.); luizabastosalves@gmail.com (L.B.A.); 2 NIHR King’s Clinical Research Facility and SLaM Biomedical Research Centre, King’s College Hospital, London SE5 9RS, UK; 3 Department of Neurology, University of California Los Angeles, Los Angeles, CA 90095, USA

**Keywords:** migraine, glutamate, excitotoxicity, genetics, NMDA, AMPA, kainate, mGluR, iGluR

## Abstract

Recurrent and intense headache is a well appreciated cardinal feature of migraine, a common and incapacitating neurological disorder. Often, there are associated canonical sensory abnormalities, such as light and sound sensitivity, as well as associated nausea. Given this phenotype of disordered sensory processing and, in a third of patients, the phenomenon called aura accompanying migraine attacks, it has been suggested that the pathophysiology of migraine is likely to involve glutamate, the main excitatory neurotransmitter in the central nervous system (CNS). Glutamate plays a role in nociception, central sensitization, and cortical spreading depression (CSD), three processes that are deemed important in migraine biology. With an emphasis on the therapeutic potential of targeting various glutamate receptors in migraine, this review will discuss the currently available literature and emerging findings on the role of targeting glutamatergic pathways for the treatment of migraine. A thorough literature review was carried out on the functions of both metabotropic glutamate receptors (mGluRs), and the ionotropic glutamate receptors (NMDA, AMPA, and kainate) in migraine pathogenesis. The ever-present need for new treatments, the role of glutamate in the migraine aura phenomenon, and the consequences of monogenic migraine mutations on mediating prolonged, complex, or permanent aura are all discussed, culminating in a suggestion that glutamatergic targeting may hold particular promise in the management of migraine aura. There are plausible roles for metabotropic receptors in regulating pain processing in important migraine-related brain structures, like the thalamus and trigeminal nucleus. Similarly, ionotropic receptors contribute to excitatory neurotransmission and neuronal hyperexcitability. Recent studies have shown preclinical and early clinical results for treatments targeting these receptors, but there are still significant issues with treatment response, including drug transport, side effects, and efficacy. With ongoing and emerging discoveries in the field, there is increasing promise of new migraine medications targeting glutamate receptors. For bench to bedside translation in this area, continued study of the molecular basis of migraine, receptor subtypes, and exploration of potential drug delivery methods are needed.

## 1. Introduction

Migraine is a common and disabling neurological condition, affecting a large portion of the population worldwide [1]. A systematic analysis carried out in 2016 for the Global Burden of Disease Study estimated the global prevalence of migraine to be 1.04 billion [2]. Migraine is characterized by recurrent episodes of intense headache, accompanied by sensory abnormalities, such as increased sensitivity to light and sound [3]. The condition can significantly negatively impact day-to-day activities. A study conducted in 2016 investigated the burden of migraine on the patient, his/her family life and relationships, and looked at 24 items across six domains, including missed/cancelled family events and negative financial impact, and found that all six domains were significantly impacted by migraine. The burden of the disease highlights a substantial need for new, effective and targeted therapeutic approaches, as many patients still report insufficient efficacy, contraindications, or intolerable side effects with currently available therapies [4]. Despite the recent emergence of targeted therapeutics, namely those acting on the calcitonin gene-related peptide (CGRP) pathway [5], there remains a significant unmet therapeutic need amongst those with migraine. Not all patients respond to these therapies, and in particular those with frequent or problematic aura remain one underserved group. Some of these patients will carry a monogenic migraine mutation, predisposing them to increased glutamatergic sensitization and cortical hyperexcitability [6]. Whilst insights into the mechanisms of migraine have been gleaned from these disorders to date, unfortunately these have not thus far been translated into treatment options.

Glutamate is a prospective promising target for new migraine treatment research, given its essential role in brain functions, such as pain modulation and nociceptive sensitization, and its interactions with the opioid system, and with sensory signal transmission [7]. Peripheral glutamate release activates N-methyl-D-aspartate (NMDA) receptors located on the meningeal afferents of the trigeminal nerve, if magnesium is displaced from the NMDA receptor [8]. Trigeminovascular nociceptive traffic in the trigeminocervical complex (TCC) is activated by glutamate locally [9]. Some portion of that effect is mediated by the NMDA receptor [10,11], some by the kainate receptor [12] and some by the α-amino-3-hydroxy-5-methyl-4-isoxazolepropionic acid (AMPA) receptor [13]. Moreover, metabotropic–mGluR5 receptors also play a role in the trigeminovascular system [14]. Centrally, glutamate can promote cortical hyperexcitability and cortical spreading depression (CSD), the neurophysiological correlate of migraine aura [15,16]. Glutamatergic neurotransmission can also promote the production and release of other neurotransmitters and neuropeptides involved in migraine, such as CGRP, further sensitizing meningeal nociceptors and contributing to central sensitization, a process involved in migraine, which is thought to contribute to allodynia, and may contribute to a reduced treatment response [17].

**Figure 1:**
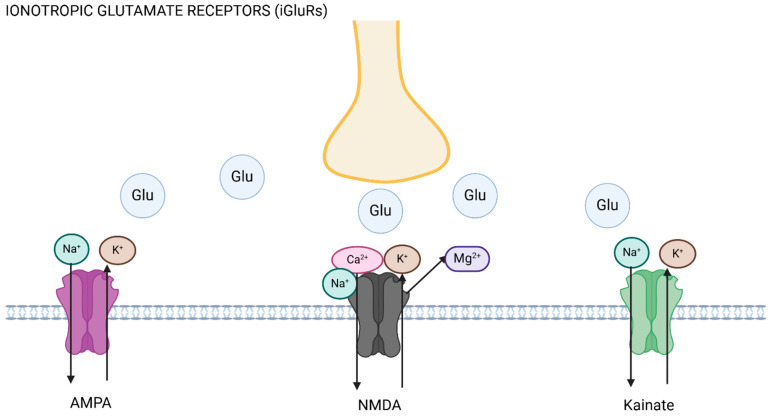
Ionotropic glutamate receptors (iGluRs). The axon (yellow) releases glutamate (Glu) upon excitation, promoting ionic flux depending on the receptor activated. AMPA and kainate receptor activation leads to inward flux of sodium (Na^+^) into the postsynaptic cell, and efflux of potassium (K^+^), whilst NMDA receptor activation leads to displacement of magnesium (Mg^2+^) and sodium and calcium (Ca^2+^) entry into the cell. Figure made using Biorender.com.

**Figure 2:**
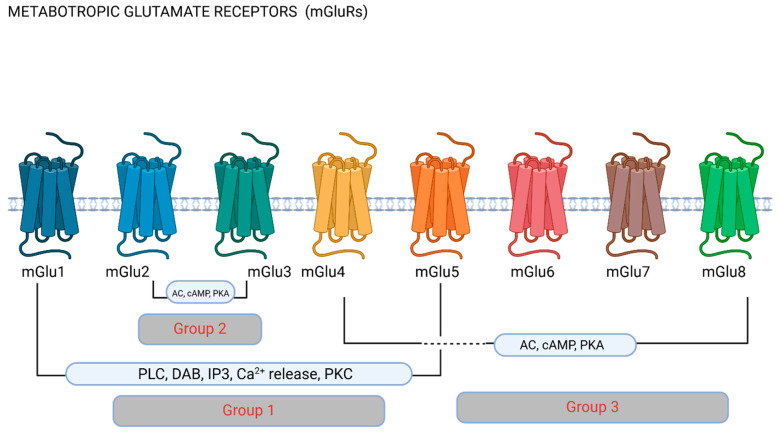
Metabotropic glutamate receptors (mGluRs). Metabotropic receptors are classified into groups depending on their pharmacology and function based on downstream cellular pathways. Group 1 receptors have been most commonly researched in migraine, and stimulate phospholipase C (PLC) and the hydrolysis of phosphatidylinositol 4,5-biphosphate (PIP2), producing inositol (1,4,5)-triphosphate (IP3) and diacylglycerol (DAG). IP3 diffusion onto the endoplasmic reticulum promotes Ca^2+^ release to the cytoplasm. Group II and III mGluRs inhibit adenylate cyclase (AC), therefore affecting downstream mechanisms via cyclic adenosine monophosphate (cAMP) and phosphokinase A (PKA). Figure made using Biorender.com.

Ionotropic glutamate receptors (iGluRs) and metabotropic glutamate receptors (mGluRs) are the two main receptor classes through which glutamate mediates its action [18]. These groups are further divided into NMDA receptors, AMPA receptors, and kainate receptors, which are the three primary subtypes of iGluRs, and these are ligand-gated ion channels [19]. These receptors are crucial to fast excitatory neurotransmission, as they play key roles in synaptic plasticity, long-term potentiation, and excitotoxicity [20] (see Figure 1). Metabotropic glutamate receptors are G-protein-coupled receptors, involved in regulating neuronal excitability and neurotransmitter release, as well as brain development and recovery after brain injury [21,22,23]. mGluRs are further organized into three categories according to the receptor structure and function based on second messenger systems. Group I includes mGlu1 and mGlu5, Group II is composed of mGlu2 and mGlu3, and Group III, the larger of the three, includes mGlu4, mGlu6, mGlu7, and mGlu8 [24] (see Figure 2). Group I receptors are largely postsynaptic and activate phospholipase C and thus increase neuronal excitability, mediating slow excitation [25], and presynaptic receptors in this group activate protein kinase C and promote glutamate exocytosis [26]. Group II and III receptors are largely located presynaptically and reduce glutamate release through adenylyl cyclase activation [27], thus mediating slow inhibition. Different groups of mGluRs therefore have distinct effects on neuronal activity, and mGluRs can have either pro-nociceptive or anti-nociceptive functions [28]. The interactions of different receptors can also lead to modulatory effects on pain processing. Through all the receptors, glutamate excitotoxicity can be triggered, i.e., neuronal over-excitation culminating in cell apoptosis or cell death, caused by excessive glutamate increasing cytoplasmic intracellular calcium [29].

Even though iGluRs have more commonly been historically researched with regards to disorders of the central nervous system, there is recent growing evidence that mGluRs may play a key role in migraine [30], neurodegenerative disorders [31] and other neurological conditions [25].

This review examines the functions of both ionotropic and metabotropic glutamate receptors in migraine and the possible therapeutic benefits of targeting these receptors, with a special focus on the mGluRs, which are less widely explored in current literature. We also evaluate current migraine pathophysiology and pharmacological research, with the goal of a better understanding of the pathogenesis of the disorder, as a means to advancing the development of more efficient therapies.

## 2. Glutamate Receptors

Glutamate plays a crucial role in neuronal activity due to its action via both ionotropic (iGluRs) and metabotropic (mGluRs) receptors. While mGluRs are G-protein-coupled receptors that run slower, longer-lasting synaptic responses, iGluRs are ligand-gated ion channels that mediate rapid synaptic transmission [32]. Therefore, the robust function of both classes of glutamate receptors is required for physiological brain activity.

### 2.1. iGluRs: NMDA, AMPA and Kainate Receptors

NMDA, AMPA, and kainate receptors are the three main classes of ionotropic glutamate receptors (iGluRs), according to their ligand specificity [33] (see Figure 1).

NMDA receptors are glutamate-gated ion channels, highly permeable to Ca^2+^, and well-known for their function in long-term potentiation (LTP) and synaptic plasticity due to changes in receptor expression [34,35]. By allowing Ca^2+^ influx, these glutamate receptors trigger several intracellular signaling pathways that increase synaptic strength and facilitate neuronal adaptations [36]. In the context of migraine, NMDA receptor activity is associated with neuronal hyperexcitability and the onset of cortical spreading depression (CSD), both of which are also mediated by increased Ca^2+^ influx [37]. Excessive NMDA receptor activation can potentially cause excitotoxicity, leading to neuronal damage, sustained nociceptive activation, and central sensitization [38,39].

Synaptic plasticity and fast excitatory signaling responses within the CNS depend on AMPA receptors, which mediate rapid neuronal depolarization [40]. These receptors are activated by glutamate exclusively and are primarily permeable to Na^+^ and K^+^, but also to Ca^2+^ to a lesser degree; all of these contribute to their role in synaptic plasticity, especially in short-term responses. During migraine, AMPA receptors play a role in the rapid depolarization of cortical neurons, which promotes the spread of CSD [41]. Their activation is also closely associated with the perception and amplification of painful stimuli, which can lead to central sensitization [42,43]. AMPA receptors also lower CSD threshold and facilitate trigeminovascular traffic [16].

Kainate receptor functions are not as well understood when compared with the other iGluRs; it is known that they mediate postsynaptic excitability and presynaptic regulation of neurotransmitter release [44]. Their presence in important migraine-related brain areas, such as the trigeminal nucleus caudalis (TNC) [45] and the somatosensory cortex [46], suggests possible functions in regulating excitatory impulses and promoting neuronal sensitization in migraine. Preclinical studies have shown that they may play a modulatory role via pro- and anti-nociceptive functions in migraine, and via interactions with other pathways important in migraine biology, such as the CGRP pathway [12,47].

### 2.2. mGluRs: Group I (mGlu1 and 5), Group II (mGlu2 and 3) and Group III (mGlu4, 6, 7 and 8)

mGluRs are G protein-coupled receptors, which use second messenger and intracellular signaling pathways to control synaptic plasticity and neuronal excitability [21]. These receptors have a slower and modulatory response to glutamate binding when compared with ionotropic receptors. They are categorized into three classes according to their signal transduction processes, pharmacological properties, and sequence similarity (see Figure 2).

Primarily found postsynaptically in dendritic spines, Group I (mGlu1 and mGlu5) receptors act by activating phospholipase C (PLC), thereby activating protein kinase C (PKC) and increasing intracellular calcium levels and augmenting neuronal excitability. Group I mGluRs are thought to be pronociceptive and participate in pain facilitation [48]. Group I receptors are also important in LTP in the context of synaptic plasticity, and mGlu5 is involved in synaptogenesis and dendritic spine remodeling [49] and may be involved in pro-inflammatory effects mediated by the release of pro-inflammatory cytokines, including substance P and CGRP [50].

Most Group II (mGlu2 and mGlu3) receptors are found presynaptically, where they suppress the release of glutamate and other neurotransmitters by blocking adenylyl cyclase activity and lowering cyclic adenosine monophosphate (cAMP) levels. Therefore, this group is involved in the maintenance of excitatory balance. These receptors are thought to be antinociceptive and provide protection by reducing excessive excitatory signals and preventing excitotoxicity [51].

More widely expressed throughout the central nervous system (apart from mGlu6), Group III (mGlu4, mGlu6, mGlu7, and mGlu8) receptors are located presynaptically, mainly in the descending pain modulation system [52]. They can also be considered as involved in fine-tuning the cell state by reducing neurotransmitter release (both glutamate and GABA) and modulating nociceptive information [53,54,55].

## 3. Glutamate in Migraine

Glutamate excitotoxic pathways are believed to be the route via which mGluRs are involved in migraine mechanisms [30], as they can result in neuronal hyperexcitability and persistent pain, thereby increasing migraine intensity and chronification [12,13]. Important migraine-related brain areas involved in the transmission and processing of pain signals express these receptors, including the thalamus, trigeminal ganglion (TG), and TNC [14]. Similarly, neuronal sensitization and excitatory neurotransmission, both involved in migraine processing, are also mediated by ionotropic receptors and provide a plausible explanation for how this class of receptors may also be involved in the complex pathophysiology of migraine [15].

### 3.1. Glutamate Levels in Migraine

There is some, albeit inconsistent, evidence of raised plasma, salivary and CSF glutamate levels in migraine, both during and outside of a migraine attack [56,57,58,59,60,61,62,63], with a suggestion that commonly used migraine preventive agents lacking a specific glutamatergic mechanism can reduce plasma glutamate levels in migraine [64].

### 3.2. Imaging Suggestion of Altered Glutamate Levels in Some Brain Regions in Migraine

Imaging studies in humans using spectroscopy have suggested that glutamate may be increased in some regions of the brain in migraine, such as the occipital cortex (from where visual aura is generated) [65,66]. A recent study suggested reduced glutamatergic functional connectivity in the anterior cingulate cortex in patients with visual snow syndrome compared to healthy controls and patients with migraine [67]. This syndrome is an increasingly recognized co-morbidity of migraine (and was previously thought to represent a persistent visual aura) and is thought to arise from visual cortex dysfunction. Interestingly, the opposite findings with regards to occipital cortex glutamate are seen in pediatric studies of migraine, where occipital glutamate seems to decrease ictally [68,69]. A subsequent study failed to replicate the findings that glutamate may be increased in the visual cortex [70], and another study showed no difference in glutamate in the pons (another important brain region in migraine) during the acute migraine attack [71].

### 3.3. Oral Ingestion of Glutamate Can Provoke Headache

Ingestion of monosodium glutamate (MSG) has been shown to provoke headache in humans [72,73]. Supportive studies have also been published in animal models, suggesting NMDA-receptor mediated roles of MSG in trigeminal pain behaviors, associated with raised plasma glutamate and CGRP, with sumatriptan or naproxen sensitivity [74], or sensitivity to co-administration of a peripheral anti-NMDA receptor antagonist [75].

### 3.4. Neuro-Anatomical Localisation of Glutamate Receptors

All of the glutamate receptors are located in the superficial laminae I and II of the trigeminocervical complex (TCC) [76], an area of importance in migraine, responsible for merging the peripheral and central afferents of the trigeminovascular pathway. Glutamatergic neurotransmission in this area is therefore likely to be implicated in modulating pain processing in migraine. Glutamate receptors are also located in other brain areas important in migraine biology, including the trigeminal ganglion, thalamus and hypothalamus and periaqueductal gray (PAG) [77,78]. Interestingly, recent functional imaging evidence suggests somatotopic organization of the PAG, and that functional connectivity downstream to the TCC is likely involved in migraine and other headache disorders [79]. There may, therefore, be dermatome-specific downstream nociceptive processing from the PAG to the TCC, and glutamate may be involved in this.

### 3.5. Migraine Drugs Acting on Glutamatergic Neurotransmission

Triptans were, until recently, the only headache-specific abortive agents available for the acute treatment of migraine. They work as agonists at the 5HT_1B/1D_ receptors and may also partially act through modulating glutamate due to co-localization of their receptors in trigeminal ganglia [80]. Targeting of glutamate receptors has been explored in migraine, given the animal model suggestions that these receptors may modulate trigeminovascular nociception [81], as well as cortical spreading depression [82].

### 3.6. Migraine Genetics

Genetics research has been able to clarify the link between glutamate signaling and susceptibility to migraine [35]. Numerous gene polymorphisms linked to migraine, especially those that affect glutamatergic pathways, have been found [36]. Mutations in genes that encode glutamate receptor subunits, such as NMDA and AMPA, have been linked to pathological receptor function and increased susceptibility to migraine [37,38].

A clear example of how genetic changes can affect glutamate signaling is familial hemiplegic migraine (FHM), a rare monogenic migraine subtype. FHM is an inherited autosomal dominant genetic condition and is classified into three types according to the identified genetic mutation: FHM1, FHM2, and FHM3 [39]. CSD is one of the key mechanisms involved in the pathophysiology of FHM because genetic mutations in all three types of the disease cause disruption of ion gradients, synaptic transmission and regulation of cerebral blood flow.

Mutations in the CACNA1A gene cause FHM1; this gene encodes a P/Q-type calcium channel [40,41]. Mutations in the ATP1A2 gene cause FHM2; this gene encodes the alpha 2 subunit of Na^+^/K^+^ ATPase [42,43]. SCN1A mutations are responsible for FHM3; this gene encodes the Na_v_1.1 voltage-gated sodium channel [44]. All three types of the condition might cause aberrant glutamate release [45] or reduced glutamate uptake by astrocytes [46,47]. Aberrant glutamate release is present in FHM1 due to a dysregulated influx of Ca^2+^ at the presynaptic membrane [41]. In FHM2, there is decreased glutamate uptake because the astrocytes rely on a Na^+^-dependent system to clear out glutamate from the synaptic cleft [43]. The accumulation of glutamate extracellularly enhances neuronal excitability, leads to excitotoxicity, and makes affected people more susceptible to CSD and migraine symptoms.

PRRT2 and CK1δ mutations, both associated with migraine and other neurological manifestations, may also involve altered glutamatergic signaling in mediating their clinical phenotypes [83,84].

### 3.7. Kynurenines

Kynurenines are produced by the catabolism of tryptophan, an amino acid, which is the basis for the synthesis of serotonin and melatonin [85]. These products have direct effects on glutamate receptors and, through this role, kynurenic acid in this metabolic pathway may be implicated in altering glutamatergic neurotransmission in migraine [86,87,88,89]. There is evidence that the kynurenine pathway may be involved in central sensitization [90] and in CSD [91] in animal models, and these are two of the recognized processes in migraine biology. CSD can be inhibited by systemic administration of L-kynurenine in a rat model [91]. In addition, there is emerging evidence that neuropeptidergic pathways involved in migraine, such as those involving CGRP and pituitary cyclase activating polypeptide 38 (PACAP38), may interact with the kynurenine pathway in migraine, and therefore pose a potential therapeutic substrate [89,92].

### 3.8. Cortical Spreading Depression (CSD)

Cortical spreading depression (CSD) is accepted as the neurophysiological correlate of migraine aura, characterized by depolarization of glial cells and neurons, associated with an increase in extracellular K^+^, a reduction in extracellular Na^+^, and significant fluxes in other ions, ultimately leading to cellular swelling, which could contribute to further changes in the extracellular space [93].

The involvement of glutamate in CSD processes has been postulated as facilitating CSD after its initiation, and therefore leading to continued neuronal excitation, largely via NMDA receptors [94], and activation of trigeminal afferents. Supporting this, there is evidence that NMDA receptor antagonists, such as memantine [95] and ketamine [96], can inhibit CSD, and can be useful clinical migraine treatments, particularly in migraine with aura [97,98,99,100,101,102]. Interestingly, despite the reliable ability of NMDA receptor antagonism with MK-801 (dizoclopine) to block CSD and TCC firing in animal models [13,103], the same effect is not seen on CSD with other efficacious anti-migraine agents used in clinical practice [103]. There may therefore be unique roles of glutamatergic pathways in aura compared to in headache.

### 3.9. Glutamate in Nociception and Central Sensitisation

Noxious stimulation in animal models causes an increase in glutamate concentration in important migraine structures, like the thalamus [104] and trigeminal nucleus [105]. Additionally, stimulation of extracranial structures, like the middle meningeal artery or superior sagittal sinus in the dura mater, which are known to be painful in animal models of migraine, causes facilitation of nociceptive neuronal fibers within the TCC [13,81].

### 3.10. Glutamate and Nitric Oxide Mechanisms of Vasodilatation

Glutamate administration promotes the release of cyclic guanosine monophosphate (cGMP) in cerebellar cultures via NMDA receptors [106]. Nitric oxide synthase (NOS) inhibitors and NO scavengers can inhibit this rise in cGMP levels, suggesting the role of NO in this pathway, likely downstream of NMDA receptors [106]. NMDA receptor activation induces NOS and promotes calcium entry into the cell [107]. Endothelial NMDA receptor activation can also induce NOS and promote NO release and vasodilation [108]. Cerebral and meningeal vasodilatation has been accepted as part of the migraine process, and NO is thought to play a part in this [109].

## 4. Current Evidence for Glutamate-Targeted Therapies in Migraine

### 4.1. Ionotropic Receptor-Targeted Therapies

#### 4.1.1. NMDA Receptor Antagonists

NMDA receptors, among other glutamate receptor types, are located in the trigeminal nucleus caudalis (TNC) [76], an important brain region in migraine. Pre-treatment with intravenous administration of an NMDA receptor antagonist, MK-801, attenuates neuronal activation in the TNC in response to chemical activation of corneal nociceptors in rodents [110]. Similarly, MK-801 could also inhibit neuronal traffic in the TCC in response to painful electrical stimulation of the superior sagittal sinus in a cat model [13]. The same agent may also have effects on pain modulation via other brainstem areas [111]. NMDA receptors play an important role in pain sensitization mechanisms [38,39,112,113], and administration of MK-801 or another antagonist, AP7, inhibits peripheral inflammation and electrical stimulation-induced sensitization in rodents [112,113]. Such studies have led to interest in NMDA receptor-mediated mechanisms in migraine, given the importance of sensitization in migraine in its chronification and treatment [114], and in medication overuse headache [115]. There is also preclinical evidence for the potential role of NMDA receptor antagonism in migraine therapeutics based on the effects of agents like magnesium and memantine on an animal model of trigeminovascular nociception [81], and of ketamine in a CSD model [116].

Magnesium is a blocker of NMDA receptors, amongst its actions on other receptor types. It is often used as a migraine treatment without strong evidence, based on its nutraceutical effect and generally acceptable tolerability.

Ketamine, a non-competitive NMDA receptor antagonist, modulates glutaminergic signaling. It has been shown in a clinical trial to reduce aura severity but not duration in prolonged aura (intranasal administration) [101], and in an open label study to reduce severity and duration of aura in FHM patients in a small cohort [102]. Randomized controlled trials of intravenously administered ketamine in Emergency Departments have failed to show efficacy [117,118]. Case series, retrospective analyses and open label studies exploring the use of ketamine for the treatment of refractory migraine also show conflicting results [119,120,121,122,123]. A meta-analysis has suggested that further randomized controlled trials are needed to establish the role of ketamine in migraine management [124]. A planned multi-center trial protocol was published in 2023 for such a trial and the results are awaited [125].

Memantine is a non-competitive, low-affinity NMDA receptor antagonist which interacts with the Mg^2+^ binding site to prevent excessive excitation while preserving normal function. Therefore, memantine has neuroprotective potential properties stemming from its ability to decrease glutamate excitotoxicity [126]. Although it is currently used mainly to treat other neurological diseases, especially Alzheimer’s, it has shown promise in preventing chronic migraine, with a favourable side effect profile making the drug largely well tolerated [100]. A 12-week randomized, double-blind, placebo-controlled study of 60 patients with migraine with aura published in 2016 showed a 62% reduction in the frequency of migraine attacks when compared with the placebo group, who experienced a 17% reduction [100]. Another study showed supportive results, with 85.7% of the patients in the memantine group achieving a reduction in migraine frequency of ≥50%, compared to 51.7% in the placebo group [98].

#### 4.1.2. AMPA Receptor Antagonists

AMPA receptors co-localize with NMDA receptors in the TCC [76], and the two receptor subtypes functionally interact in pain and sensitization pathways [127]. A specific AMPA receptor antagonist, GYKI53466, could inhibit nociceptive TCC neuronal activity [13], and AMPA/kainate antagonism could reduce neuronal activation in the TCC in response to capsaicin in an animal model [128]. This effect is deemed likely to be due to AMPA receptor antagonism, as selective kainate receptor antagonism failed to produce the same result [128]. An AMPA/kainate receptor antagonist, LY293558, has demonstrated efficacy and tolerability in the acute treatment of migraine when intravenously administered in 44 patients with migraine, compared to subcutaneous sumatriptan or placebo, in a randomized triple-blind, parallel group, double dummy multicenter trial [129]. Similarly, another AMPA receptor antagonist, BGG492, has provided randomized controlled evidence for safety, efficacy and tolerability after a single dose when trialed for acute migraine treatment, compared to both sumatriptan 100mg orally and placebo [130], with 2 h pain freedom similar to that of sumatriptan.

#### 4.1.3. Kainate Receptor Antagonists

Kainate receptors are located in peripheral (trigeminal ganglion) [131] and central (TNC and thalamus) [12] areas of interest in migraine. The receptors are located both pre- and post-synaptically and can therefore exert their function in a variety of ways, from regulation of neurotransmitter release to neuronal current modulation [132]. In animal models, kainate receptor activation causes pain behaviors and sensitization [133]. A kainate receptor antagonist LY466195 [134] inhibits nociceptive TCC neuronal activity in an animal model [12]. Activation of iGluR5 kainate receptors with the selective agonist iodowillardiine could inhibit neurogenic dural vasodilatation in an animal model, likely through inhibition of prejunctional release of CGRP from trigeminal afferents [47]. Taken together with recent clinical studies, the data reinforce CGRP mechanisms in primary headache disorders, and demonstrate a novel role for kainate receptor modulation of trigeminovascular activity, with both pro- and anti-nociceptive functions. Topiramate, which is well-known as a broad-spectrum anti-epileptic medication, in randomized clinical trials has demonstrated effectiveness in preventing migraine [135,136,137,138,139,140,141]. Topiramate is primarily a GABAergic modulator, but its therapeutic effect in migraine treatment is achieved by its concomitant AMPA and kainate receptor inhibition in the trigeminovascular system, where it decreases neuronal hyperexcitability [142].

In an animal model, a kainate receptor antagonist, LY466195, did not have a vascular effect on alpha-CGRP, capsaicin or electrically-induced vasodilatation in a rat model (whereas ketamine and MK-801, working together as another NMDA receptor antagonist, did have some vascular effects) [143], so any effects of such agents in migraine therapeutics is likely to be mediated centrally rather than via the peripheral vasculature.

### 4.2. Metabotropic Receptor-Targeted Therapies

Group I mGluRs are generally the most widely researched in migraine, as they are largely located postsynaptically, and receptor activation increases neuronal excitability and promotes nociception, whereas Groups II and III are considered antinociceptive through presynaptic modulation of glutamate release [32].

#### 4.2.1. Group I mGluR Antagonists

These agents inhibit receptor activity through prevention of glutamate binding to the receptor, thereby reducing neuronal excitation. Within this group, the most widely researched receptor is mGlu5, based on its localization on trigeminal sensory afferents within the dural vasculature [144] and in the trigeminal ganglion [145] and TCC [146], and because of its role in several other pain states, such as inflammatory and neuropathic pain [147,148]. mGlu5 may have roles in pain sensitization and chronification [147] and may also interact with other receptor systems and pathways, such as the opioid pathway [149], and this too has been implicated in migraine biology in mediating sensitization [150].

Fenobam is a selective, potent and non-competitive mGlu5 antagonist that was first used as an anxiolytic [151] but, later, was found to be an analgesic in rodents, and to reduce sensitization in humans [152,153]. Its use was limited due to undesired side effects, such as cognitive impairment and sedation [152]. Another group of antagonists at mGlu5, termed selective negative allosteric modulators (NAMs), meaning that they bind to a site that differs from the glutamate binding site and thereby modify the receptor’s configuration, have also been investigated as anti-migraine agents. The NAM mGlu5 antagonist ADX10059, specifically developed for migraine prevention with a focus on migraine with aura [154], has been shown to attenuate vasodilator responses to meningeal stimulation in a rodent model, in a manner comparable to naratriptan, and to reduce trigeminocervical neuronal firing in response to dural stimulation, thereby demonstrating both peripheral and central anti-migraine effects. A double-blind placebo-controlled parallel group clinical trial in human subjects showed that the agent was superior to placebo in achieving the primary outcome of pain freedom at 2 h. The agent was well tolerated, although dizziness was not uncommon [14]. Unfortunately, a subsequent study identified hepatotoxicity as a concern with prolonged use of the agent [155], so all further trials and drug development were stopped. Other negative allosteric modulators of mGlu5 have not been demonstrated to have hepatotoxic effects in movement disorder studies, so this may still be a useful avenue in migraine therapeutics [156].

The other receptor in this group, mGlu1, is relatively unexplored in migraine biology. Like mGlu5, it is located in important areas of interest and also has roles in facilitating nociceptive processing through the TCC, and this processing may be modulated by estrogens. Compared to mGlu5, the anatomical localization of mGlu1 receptors seems to be much higher in the central pain processing pathway, including in the thalamus, where they may interact with NMDA-mediated neurotransmission to alter neuronal activity.

#### 4.2.2. Group II and III mGluR Agonists

Activation of both Group II (mGlu2 and mGlu3) and Group III (e.g., mGlu4, mGlu7) receptors to decrease glutamate release and therefore neuronal excitability has potential neuroprotective and anti-nociceptive effects [51,52]. In particular, Group II and III mGluRs modulate GABA inhibitory neurotransmission in thalamic areas [157]. Preclinical research lacks any studies specific to migraine, although there is some evidence for an agonist to Group II mGluRs, LY379268, in chronic pain models and sensitization in rodents [158]. An mGluR2 potentiator and cysteinyl leukotriene 1 antagonist, LY2300559, has proof of concept evidence for efficacy as migraine prevention in a randomized double-blind placebo-controlled trial (abstract only published) [159].

## 5. Other Therapies

There is some evidence for nutraceutical agents, like magnesium as discussed previously, but also riboflavin, coenzyme Q10 and omega 3 fatty acids, acting on glutamatergic pathways, and also providing some evidence in migraine treatment [160].

## 6. Conclusions

There is evidence spanning several decades for the role of glutamate in migraine, and previous and emerging evidence for glutamate as a potential therapeutic target for migraine. This includes animal model and human imaging and genetic studies. Glutamate offers a unique target for migraine with aura, given this group of patients remain underserved by currently available therapies, when the aura specifically is troublesome more than, or in addition to headache (see Table 1). However, preclinical and clinical research has unfortunately to date not led to pharmacological development in migraine, outside of the use of memantine targeting NMDA receptors as a possible migraine preventive therapy.

Targeting different glutamate subtypes can cause adverse effects, and mixed actions of some of the agents identified on more than one receptor type can complicate drug development in this context. The widespread distribution of glutamate receptors within the central nervous system, and the vital role of the neurotransmitter in normal neuronal function (including learning and cognition [161]), complicate therapeutic development given the potential significant risk of adverse effects. In addition, the role of some receptor subtypes, in particular the mGluR subtypes in migraine biology, remains yet to be elucidated and there is much to be learned about the roles of these different receptor types, interactions with other pain pathways, and the identification of further potential therapeutic substrates. There are, therefore, a lack of pharmacological agents targeting glutamate itself or its receptors for the treatment of migraine at present, but furthering understanding of the mechanisms of different receptor types, targeting of modulators of glutamatergic signaling, like the kynurenine pathway, and understanding means of specific receptor subtype targeting, may form plausible means in the future for glutamate to be further investigated as a migraine therapeutic substrate, ideally without the compromise of intolerable side effects.

**Table 1:** Summary of the glutamate-targeted therapies against different receptors that have been researched in migraine, with their preclinical and clinical evidence and translation to clinical practice where applicable.

Receptor Mechanism	Preclinical Evidence in Migraine	Clinical Evidence in Migraine	Effect on Clinical Practice
**Ionotropic glutamate receptors**			
NMDA receptor antagonism	MK-801 attenuates neuronal activation in the TNC in response to chemical activation of corneal nociceptors in rodents [110] MK-801 inhibits neuronal traffic in the TCC in response to painful electrical stimulation of the superior sagittal sinus in cats [13] MK-801 may also have effects on pain modulation via other brainstem areas [111] MK-801 or another antagonist, AP7, both inhibit peripheral inflammation and electrical stimulation-induced sensitization in rodents [112,113] Magnesium and memantine have effects on an animal model of trigeminovascular nociception [81] Ketamine has an effect in a CSD model in animals [116]	Magnesium [162,163,164,165] and memantine [98,100] have some randomized clinical trial evidence for efficacy in migraine treatment Ketamine has uncontrolled and open label evidence for an effect in aura [100,101,116,117,118,119,120,121,122]	Magnesium, ketamine, and memantine are used clinically in migraine management The authors do not use ketamine in their practice
AMPA receptor antagonism	AMPA receptor antagonist, GYKI53466 inhibits nociceptive TCC neuronal activity [13] AMPA/kainate antagonism reduces neuronal activation in the TCC in response to capsaicin in an animal model [128] and selective kainate receptor antagonism failed to produce the same result [128], suggesting this is an AMPA receptor-mediated effect	IV AMPA/kainate receptor antagonist, LY293558, has demonstrated efficacy and tolerability in the acute treatment of migraine compared to subcutaneous sumatriptan or placebo in a randomised triple-blind, parallel group, double dummy multicentre trial [129] AMPA receptor antagonist, BGG492, has randomised controlled evidence for safety, efficacy, and tolerability after a single dose when trialed for acute migraine treatment and compared to both sumatriptan 100mg orally, and placebo [130], with 2-hour pain freedom rates similar to sumatriptan	None of these therapies have reached clinical practice
Kainate receptor antagonism	Kainate receptor antagonist LY466195 [134] inhibits nociceptive TCC neuronal activity in an animal model [12] Activation of iGluR5 kainate receptors with the selective agonist iodowillardiine inhibits neurogenic dural vasodilatation in an animal model, likely by reducing CGRP release from trigeminal afferents [47].	Topiramate has GABAergic effects, and concomitant AMPA and kainate receptor inhibitory effects in the trigeminovascular system, where it decreases neuronal hyperexcitability [142]	Topiramate is used clinically in the treatment of migraine [135,136,137,138,139,140,141]. Caution must be exerted, especially in pre-menopausal females), due to teratogenicity risks, and effects on reducing contraceptive efficacy
**Metabotropic glutamate receptors**			
Group I receptor antagonism			
mGlu5	Fenobam, an mGlu5 antagonist, has an analgesic in rodents, and reduces sensitization in humans [152,153]. Undesired side effects such as cognitive impairment and sedation [152] limit its use NAM mGlu5 antagonist ADX10059, attenuates vasodilator responses to meningeal stimulation in a rodent model in the same way as naratriptan, and reduces TCC neuronal firing in response to dural stimulation [154]	Double-blind placebo-controlled parallel group clinical trial of ADX10059 superiority to placebo in pain freedom at 2 hours. Dizziness as a side effect was not uncommon [14] A subsequent study identified hepatotoxicity as a concern with prolonged use of ADX10059 [155], so all further trials and drug development were stopped	This therapy has not reached clinical practice due to safety concerns
Group II receptor agonism			
	Some evidence for an agonist to Group II mGluRs, LY379268, in chronic pain models and sensitization in rodents [158], but no specific migraine studies have been conducted	mGluR2 potentiator and cysreinyl leukotriene 1 antagonist, LY2300559, has proof of concept evidence for efficacy as migraine prevention in a randomised double-blind placebo-controlled trial (abstract only published) [159].	This therapy has not reached clinical practice as yet

## Data Availability

No new data were acquired for production of this manuscript.
